# Processive Pectin Methylesterases: The Role of Electrostatic Potential, Breathing Motions and Bond Cleavage in the Rectification of Brownian Motions

**DOI:** 10.1371/journal.pone.0087581

**Published:** 2014-02-04

**Authors:** Davide Mercadante, Laurence D. Melton, Geoffrey B. Jameson, Martin A. K. Williams

**Affiliations:** 1 The Riddet Institute, Palmerston North, New Zealand; 2 School of Chemical Sciences, University of Auckland, Auckland, New Zealand; 3 Institute of Fundamental Sciences, Massey University, Palmerston North, New Zealand; 4 The MacDiarmid Institute for Advanced Materials and Nanotechnology, Victoria University, Wellington, New Zealand; Universidad de Granada, Spain

## Abstract

Pectin methylesterases (PMEs) hydrolyze the methylester groups that are found on the homogalacturonan (HG) chains of pectic polysaccharides in the plant cell wall. Plant and bacterial PMEs are especially interesting as the resulting de-methylesterified (carboxylated) sugar residues are found to be arranged contiguously, indicating a so-called processive nature of these enzymes. Here we report the results of continuum electrostatics calculations performed along the molecular dynamics trajectory of a PME-HG-decasaccharide complex. In particular it was observed that, when the methylester groups of the decasaccharide were arranged in order to mimic the just-formed carboxylate product of de-methylesterification, a net unidirectional sliding of the model decasaccharide was subsequently observed along the enzyme’s binding groove. The changes that occurred in the electrostatic binding energy and protein dynamics during this translocation provide insights into the mechanism by which the enzyme rectifies Brownian motions to achieve processivity. The free energy that drives these molecular motors is thus demonstrated to be incorporated endogenously in the methylesterified groups of the HG chains and is not supplied exogenously.

## Introduction

**Figure 1:**
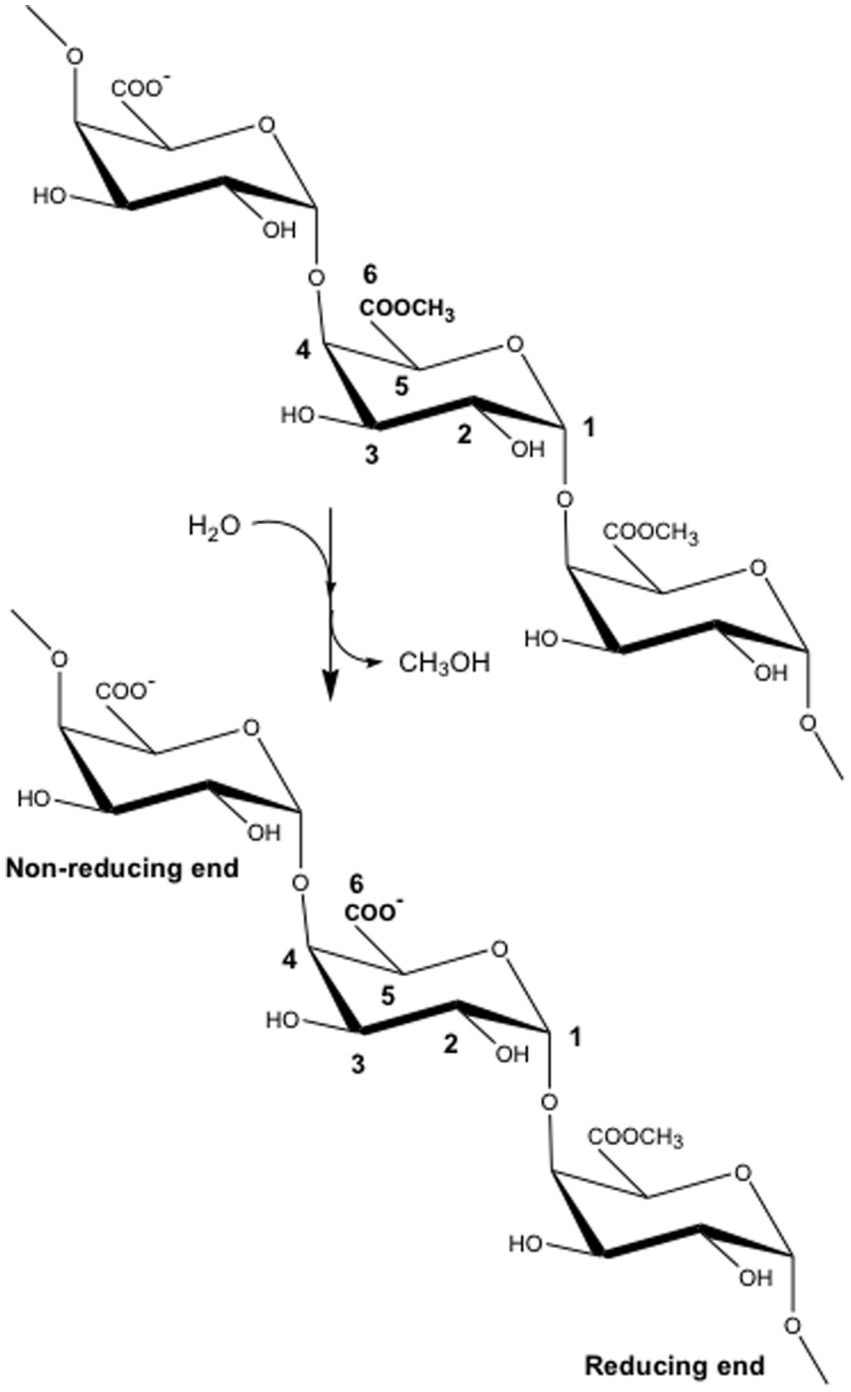
An example of the hydrolysis reaction catalyzed by pectin methylesterase (PME). A homogalacturonan trimer, with conventional atom-labelling scheme, undergoes de-methylesterification of the middle subunit.

Plant pectin methylesterase (PME) enzymes are actively involved in the re-modelling of the plant cell wall (PCW). They catalyze the de-methylesterification of the O6-methyl-galacturonate moieties that constitute the linear homogalacturonan (HG) sections of pectic polysaccharides (Fig. 1). Decreasing the degree of methylesterification (DM) modifies the physicochemical properties of the HGs, which are involved in vital physiological roles in plants [1]–[9], including the extension of the PCW, seed germination and fruit ripening. Bacteria and fungi also express their own PMEs in order to yield low-DM HG chains, whose subsequent disassembly by polygalacturonase weakens the PCW and facilitates infection [10]–[14]. The importance of PME in both eukaryotic and prokaryotic organisms is evidenced by the high number of different isoforms encoded in their genomes [15]. Intriguingly, putative PMEs have been discovered recently in the transcriptomes of such evolutionarily distant organisms as a mountain pine beetle (*Dendroctonus ponderosae*) [16] and a rice weevil (*Sitophylus oryzae*) [17], suggesting PME is perhaps of ancient lineage.

From a biophysical point of view, bacterial and plant PMEs are of particular interest for their ability to catalyze consecutive reactions along the HG chain before dissociating from it. Processivity requires the upgrade of thermal energy available in the form of Brownian fluctuations into mechanical work. Such upgrades are of fundamental interest and have been widely studied, for example in the cases of the canonical ATP-powered actin- and microtubule-associated linear molecular motors or the ion-gradient-powered rotary motors F_o_-F_1_-ATP synthase [18]–[22]. Of particular interest here is that bacterial and plant PMEs do not require ATP hydrolysis or the relaxation of ion gradients to function; instead the chemical free energy that rectifies the Brownian fluctuations is carried on the substrate itself.

**Figure 2:**
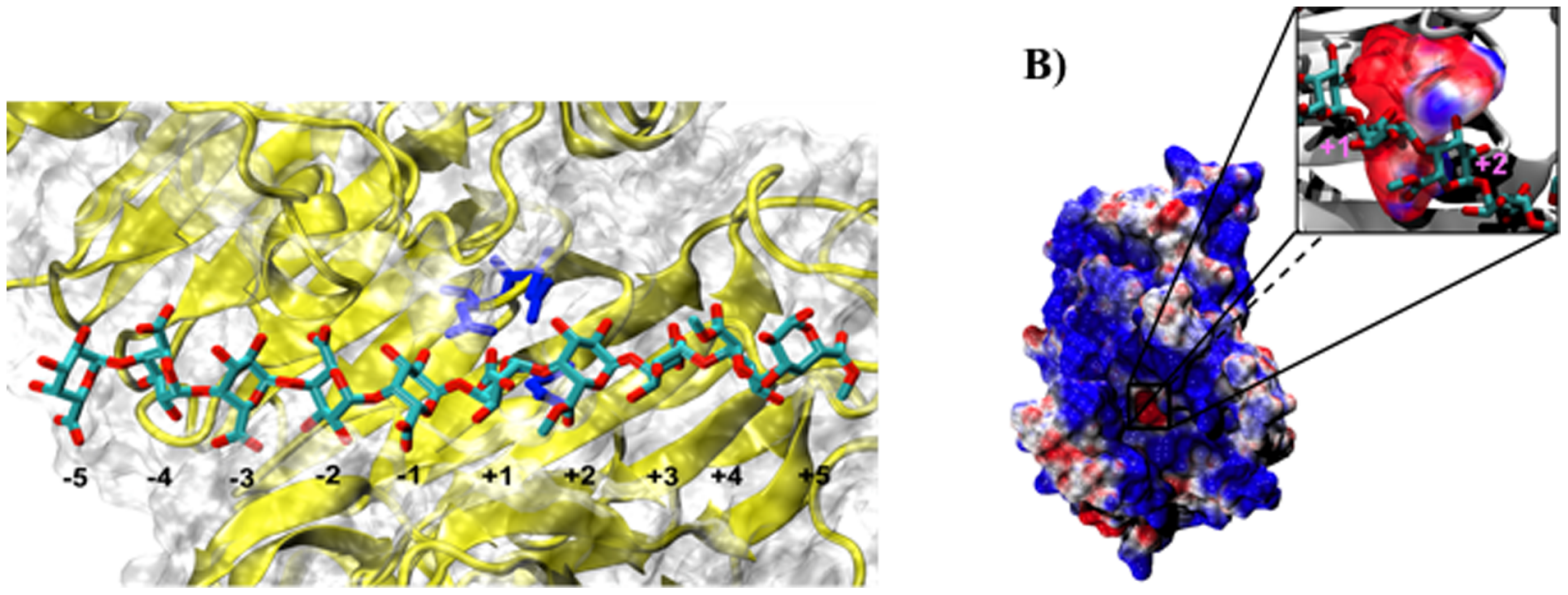
The *Ec*-PME protein. (A) Representation of the Ec-PME binding groove in an Ec-PME-HG decasaccharide complex. PME secondary structure is represented in cartoon and its surface is shown in white. The catalytic triad is represented in blue and coincides with the +1 subsite along the binding cleft. The HG decasaccharide is represented in cyan for carbon and red for oxygen atoms. (B) Electrostatic potential calculated for Ec-PME. The protein surface is colored in blue (positive) and red (negative) in the range between +3 k_b_T and −3 k_b_T (left panel). The negative patch observable in the active site is shown in close up (right panel) and is generated by the carboxylate groups of Asp178 and Asp199 that actively participate in the substrate catalysis and form an oxyanion. The oligosaccharide is shown, for clarity, in ball-and-stick representation and colored by atom type. Monosaccharide residues docked in the +1 and +2 subsites are labelled.

Structures of PMEs from the plants tomato (*Solanum lycopersicum*) [23] and carrot (*Daucus carota*) [24], and from the bacteria *Erwinia chrysanthemi* [25] and *Yersinia enterocolica* [26] have been elucidated using X-ray crystallography. These enzymes fold into a triple β-helical motif [27]. The binding interface for the HG chain, which is highly conserved, can be subdivided into subsites, each one able to bind a single monosaccharide residue of the HG chain (Fig. 2(A)). The binding groove accommodates a total of ten sugar rings. The catalytic subsite where de-methylesterification takes place is positioned in the center of the binding groove and is defined by convention as “+1”. Subsites towards the reducing end of the HG chain are numbered +2, +3, +4 and +5 starting from the catalytic subsite at +1, while those towards the non-reducing end are numbered −1, −2, −3, −4 and −5. Fig. 2(B) shows the electrostatic potential calculated for *E. chrysanthemi* PME (*Ec-*PME). The negative patch observable in the active site is generated by the carboxylate groups of Asp178 and Asp199 that actively participate in the substrate catalysis and form an oxyanion. Interestingly, X-ray crystal structure studies using four differently patterned hexasaccharide substrates have revealed that subsites −1 to −3 of the enzyme preferentially bind un-methylesterified residues, whereas subsites +1 and +3 preferentially bind methylesterified residues. The other subsites exhibit little preference [25]. Moreover, the hexasaccharides were found to adopt an alternating quasi-2_1_ screw axis conformation along the binding groove, such that the C6 substituent groups of the monosaccharide subunits, –CH_2_COOR (R = H, Me), face alternately into or away from the interior of the protein.

The extensive data base of structural information on the interaction of hexameric HG chains with *Ec*-PME allowed us to design *in-silico* variously patterned decameric HG chains that span the entire binding site. Then, by means of molecular dynamics simulations (MD) we investigated inter alia the changes in substrate-protein interactions arising from the de-methylesterification of the sugar residue in the catalytic pocket [28]. The catalytic reaction uncloaks a negative charge within the active site that results in: 1) remarkable concerted rotations of the sugar residues that remove the freshly de-methylesterified negatively-charged residue from the active site and concomitantly reorient the outward-facing methylester group of the neighboring sugar residue in the +2 site into the conformation required for processing at the active (+1) site; and 2) the complex becoming significantly more mobile as the substrate undergoes Brownian motion in a new potential [28]. Despite low expectations (given the low turnover number of the enzyme) that such Brownian walks would yield a net unidirectional sliding of the polysaccharide relative to the protein within the limited timescale accessible to us by MD, one simulation fortuitously sampled fluctuations that resulted in just such behaviour. The analysis of the electrostatic interaction potential between enzyme and substrate reported here provides new insights into the processivity of *Ec*-PME, linking the modulation of the energy landscape brought about by de-methylesterification of the substrate to the rectification of Brownian motion.

## Results

Our MD simulations carried out on the described *Ec*-PME-decasaccharide complex serendipitously sampled translocation of this decasaccharide (by one monosaccharide residue at the active site). Specifically, this substrate (designated HXM) had methylester groups towards the reducing end of the chain (binding at subsites +2 to +5) and, as such, the directionality of the translocation observed strongly suggests that such PME movements provide a mechanism for the processive de-methylesterification of the HG chain. We have previously established [28] that after de-methylesterification in the +1 subsite the methylester group of the following residue docked in subsite +2 becomes oriented (but not yet positioned) to undergo catalysis. Such conformational changes are observed within a short time (<30 ns) in all simulations on this decasaccharide (but not on other differently patterned decasaccharides). Moreover, the de-methylesterified residue at subsite +1 itself rotates away from the active site, in order to obtain a favorable orientation for subsequent accommodation at subsite −1, and thereby to preserve the alternating orientation of the most tightly held residues [28].

**Figure 3:**
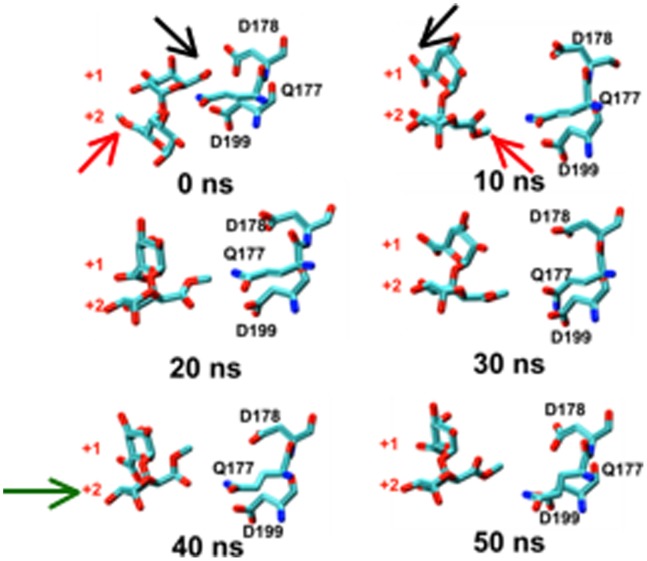
Rotation along the glycosidic bond and sliding of the substrate are the key events for the processive action of *Ec*-PME. Time-evolution of the conformational variations occurring for the catalytic triad of *Ec*-PME and two monosaccharide residues of the HXM decasaccharide, labelled by the subsite in which they were originally docked (+1 or +2). 0–10 ns: concerted approximately 180° rotation of monosaccharide residues at subsites +1 and +2. 10–30 ns: Brownian fluctuations of this disaccharide moiety and active-site side chains. 30–50 ns: movement of disaccharide moiety relative to protein bringing, a fresh carboxymethyl group (that began in subsite +2 and is still labelled as such) into the active site at +1. Carbon atoms are colored cyan, oxygen atoms are colored red and nitrogen atoms are colored in blue. Black and red arrows show the carboxylate and carboxy-methylesterified groups of the monosaccharides initially docked in the +1 and +2 subsites respectively. The green arrow in the 40 ns window signals the movement of the monosaccharide that began in the +2 subsiste into the active site (+1 subsite). Note the similarity between the final position (50 ns) occupied by the sugar ring that began in subsite +2 and the initial position (0 ns) of the residue that started in subsite +1.

Subsequent larger-scale sliding events that bring the newly rotated +2 residue into the +1 subsite are expected to be considerably slower than the rotations sampled and are governed by thermal fluctuations of the HG chain inside the binding groove. Nonetheless, the stochasticity in substrate turnover at the individual molecule level ensures that such a sliding event can be sampled at much shorter than average turnover times, as indeed occurred here (Fig. 3).

### *Ec*-PME Breathing Motions Facilitate Uni-directional Sliding of the Polysaccharide along the Binding Groove

**Figure 4:**
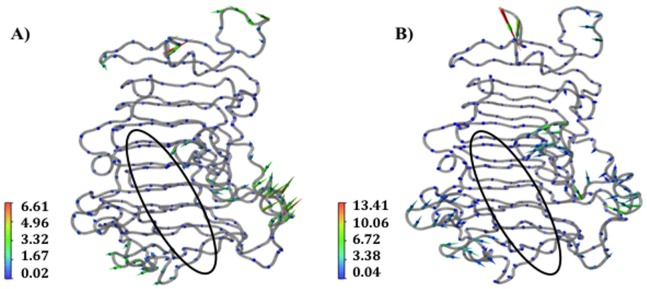
Correlated motions of the regions flanking the enzyme-substrate binding interface are crucial for the sliding of the polysaccharide along the binding cleft. Porcupine plots of the *Ec*-PME backbone in complex with the HXM decasaccharide (oligosaccharide not shown for simplicity) in the time windows 15 ns to 30 ns (A) and 30 ns to 50 ns (B). The enzyme binding-groove is indicated by the black ellipse. The represented motions account for more than 80% of the total correlated motions of the enzyme as reported for the first 8 eigenvectors along the trace of the matrix. Arrows point in the direction of the motion with length and color representing the total displacement along the sampled window. Color bars quantify the displacement in Ångstroms.

**Figure 5:**
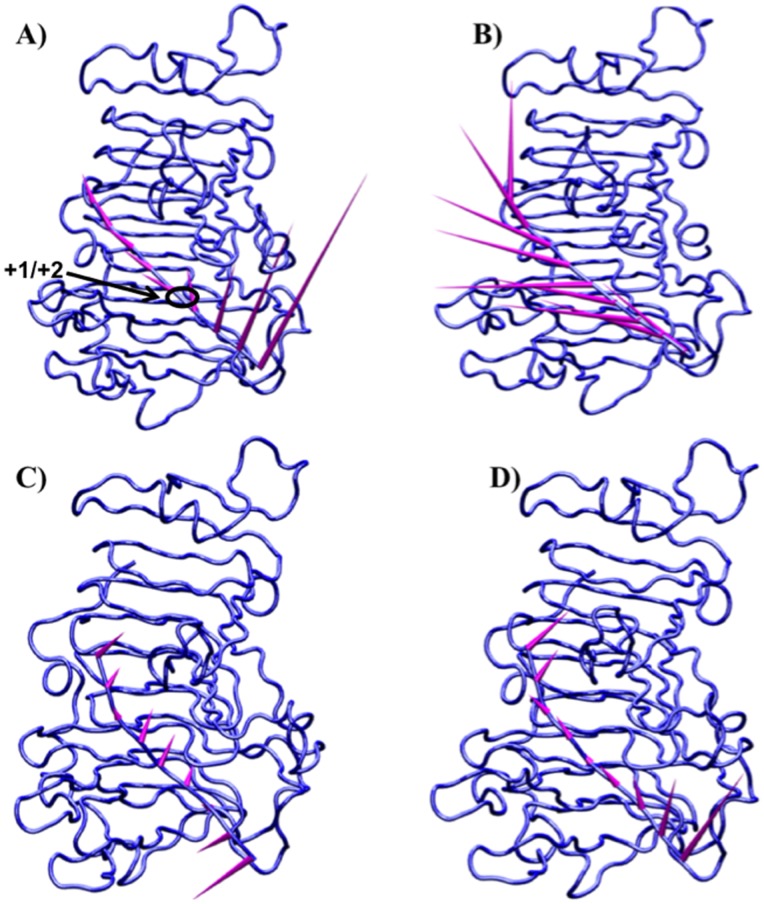
Porcupine plots of the HXM decasaccharide in complex with *Ec*-PME in the time windows 15 ns to 30 ns (A and C) and 30 ns to 50 ns (B and D). Arrows point in the direction of the motion calculated for the glycosidic bonds linking consecutive monosaccharide residues along the decasaccharide HG chain. In C and D only motions along the direction of the sliding are reported. The length of the arrows is proportional to the amplitude of the motion, which reaches a maximum of ∼10 Å for the glycosidic bond linking the pair of loosely held residues in subsites +4 and +5. Note the large component of vertical motion, consistent with the opening of the binding cleft and the loosening of binding of the HXM oligomer. Note also that the final docking of the whole oligomer with the restoration of the alternating monosaccharide residue orientations and the de-methylesterification reaction are not sampled in these molecular mechanics studies.

**Figure 6:**
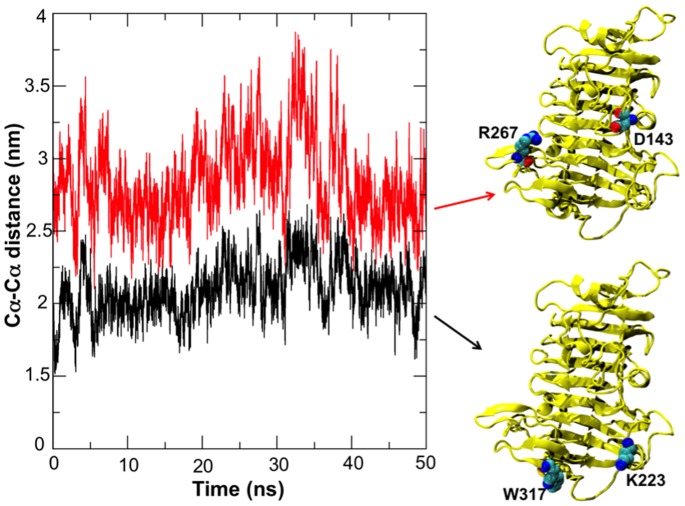
Cα-Cα distances between the residues Arg267 and Asp143 (red) and Trp317 and Lys223 (black) located at the opposite ends of the *Ec*-PME binding groove. The graph shows the distances for the simulation where a full sliding of the oligosaccharide along the binding groove is reported. In such a case, correlated motions of the two flanking regions (see also figure 3) are observed along the binding groove and contribute to the docking of a fresh methylesterified monosaccharide residue into the active site of the protein.

In addition to substrate rotations, concerted motions of protein regions flanking the binding groove also occurred. Movement of the flanking regions generated the partial release of the substrate and a concomitant partial loss of affinity for the ligand. The conformational changes observed for the enzyme itself are consistent with the different conformations of *Ec*-PME found in the X-ray crystal structures of the free enzyme or of the enzyme bound to HG hexasaccharide substrates [25] (data not shown). In the more open conformation of the protein the sliding of the HXM oligosaccharide occurs before the protein grips the decasaccharide again by returning to a more closed conformation. Interestingly, the movements of the flanking regions on both sides of the binding groove are correlated with those of the oligosaccharide, as evidenced by the principal component analysis performed on the collected trajectories of the protein (Fig. 4) and of the decasaccharide (Fig. 5). Within 20 ns, the opening of the groove is sampled, while the thermal fluctuations sampled in the stochastic walk occurring between 20 and 30 ns drive the monosaccharide residue previously docked in +2 into the active site, ready for a second catalytic event (Fig. 3). After some 30 to 40 ns, the protein once again binds the oligosaccharide more strongly. Significantly, while the opening and closing of the reducing and non-reducing ends of the protein are found to be synchronized the amplitude of the breathing is somewhat different, producing a funnelling effect that helps to facilitate the translation (Fig. 6).

### The Electrostatic Pre-organization of *Ec*-PME Binding Groove is Involved in the Sliding of the Chain

**Figure 7:**
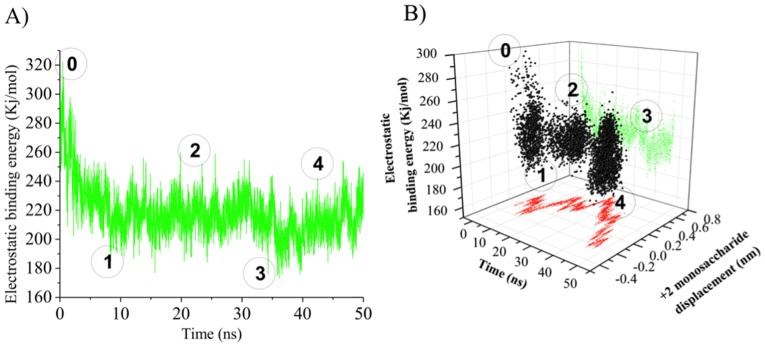
Electrostatic binding energy profile of the sliding motions of HG decasaccharide in complex with *Ec*-PME. Calculated electrostatic binding energy of *Ec*-PME in complex to the HXM decasaccharide as a function of the time (A) and movement along the binding groove of the monosaccharide residue docked in the +2 subsite (B). The circled numbers indicate different phases observable during the simulation: 0) start of the simulation; 1) end of rotation of the monosaccharide subunits docked in the subsites +1 and +2 about the glycosidic linkage, 2) stationary thermal fluctuations; 3) siting of the monosaccharide residue previously in the subsite +2 into the catalytic pocket (subsite +1) 4) continued Brownian sliding of the decasaccharide along the binding groove.

The role of electrostatic repulsion between the PME’s active-site carboxylate residues (Asp199 and Asp178) and the catalytically-formed HG carboxylate residue in driving the net unidirectional translation of the chain along the binding groove was investigated by means of continuum electrostatic calculations on the collected trajectories. The variation in the electrostatic potential of the *Ec*-PME-HG complex is shown in Fig. 7 (A).

By aligning the calculated energy profile with the conformational states of the complex along the MD trajectory (Fig. 3), the first decrease of energy can be associated with the rotations of the sugar residues as suggested (Fig. 7 (B)). The high initial electrostatic potential displayed in Fig. 7 (A) is due to the local electrostatic repulsion between the carboxylate group of the de-methylesterified monosaccharide residue docked in the subsite +1 and the carboxylate of Asp 199 in the active site (Fig. 3). This repulsion drives the rotations around the glycosidic linkages that decrease the electrostatic potential. The following stage shows the response to random thermal fluctuations, with the protein in a more open conformation, loosening its grip on the oligosaccharide as described above and illustrated in Figs. 5 (B) and 6. Eventually these thermal fluctuations result in the substrate sampling the minimum of the new potential by sliding along the chain. Indeed, a second, although less dramatic, reduction of the electrostatic potential is observed when the oligosaccharide slides along the binding groove to the extent that the sugar residue previously bound in the +2 subsite now becomes docked into the catalytic pocket, ready for the next catalytic event (Fig. 7 (A&B) & Fig. 3). Plotting the electrostatic potential as a function of the movement of the sugar residue initially docked in the +2 subsite reveals that the second potential drop indeed coincides with the movement of the chain along the binding groove (Fig. 7 (B)). That is: when the Brownian fluctuations position the sugar ring that began in subsite +2 into the active site (+1), a translation of some +0.45 nm, the electrostatic energy is minimized. It should be noted that in this simulation there is no active-site chemistry included to demethylesterify the newly- translated residue. As such Brownian fluctuations that continue to buffet the oligosaccharide can move it away from the active site again. Nevertheless, the association between the minimum in electrostatic interaction energy and the repositioning of the sugar residue in the active site is clear. Where subsequent de-methylesterification can take place the concomitant modification of the potential energy landscape constitutes a ratchet and imposes a net uni-directionality.

**Figure 8:**
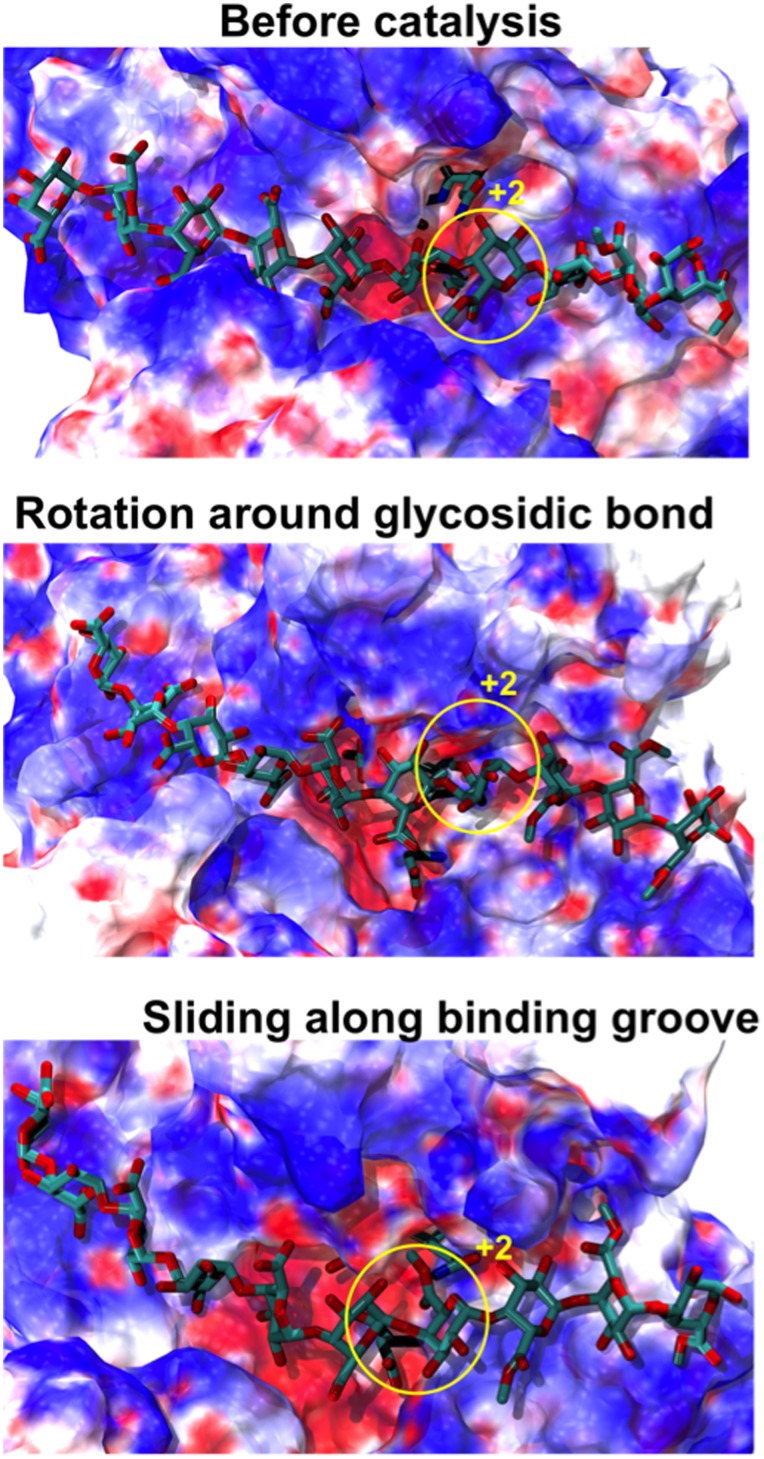
Time-evolution of the electrostatic profile for *Ec*-PME. The *Ec*-PME surface is colored by the calculated electrostatic potential of the protein in complex to the HXM decasaccharide (shown in ball-and-stick representation). The figures represent the conformations of protein and oligosaccharide before and after the catalysis, when rotation of monosaccharide subunits around the glycosidic bond and sliding of the oligosaccharide along the binding groove occur. Patches of positive electrostatic potential patches on the protein surface are represented in blue, negative patches are reported in red and neutral patches are in white. The surface is colored between −3 k_b_T (red) and +3 k_b_T (blue). The monosaccharide residue docked, at the start of the simulation, in subsite +2 is circled in yellow.

Analysis of the electrostatic potential of *Ec*-PME shows a distinctive organization of electrostatic moieties along the binding groove. A single negatively charged patch, corresponding to the active site, is visible and is responsible for the electrostatic repulsion between the de-methylesterified subunit and the oxyanion carried by Asp199 as discussed (Fig. 2(B) & 8). Conversely, basic residues flanking the binding groove surround the active site with a strongly positive potential. The distribution of charged regions is not symmetric along the groove: highly positively charged regions are more common at sites that bind the non-reducing end of the substrate (subsites −1 and −2) and more apolar regions are found at sites that dock the reducing end of the chain (subsites +1 and +3). This asymmetric electrostatic potential coincides with previous observations suggesting that the enzyme preferentially binds methylesterified residues at the subsites +1 and +3 and de-methylesterified residues at subsites −1 to −3 [25].

Monitoring the conformational variations of the decasaccharide shows that subsite −2 is primarily responsible for the biasing of potential energy landscape that the thermal fluctuations explore. Indeed, the presence of a negatively charged sugar residue at the −1 subsite is crucial to favor the processive motion of the enzyme as, after the described rotations around the glycosidic linkages, the carboxylate group of the sugar residue initially docked in subsite −1 is now oriented such that translations towards the −2 subsite expose it to a highly positive electrostatic potential.

## Discussion

The major structural difference between plant and bacterial PMEs is that the binding groove is somewhat deeper in the latter case and is flanked by longer partially-structured loops. These loops may help the bacterial PMEs avoid inactivation by proteinaceous PME inhibitors (PMEI) that are expressed by plants to finely modulate the activity of their own endogenous PMEs [23], [29]. The saddle-shaped binding groove observed for plant and bacterial PMEs resembles that for other carbohydrate-binding enzymes. Although some of these enzymes are reported to be processive [30] others are not [31]. Moreover, for the pair of enzymes endopolygalacturonase I and II, mutation at the −5 site, which is remote to the active site at +1, changed endogalacturonase I from processive to non-processive (Arg→Ser) and, conversely, endogalacturonase II from non-processive to processive (Ser→Arg) [32]. Thus, it is clear that the presence of a deep binding groove alone is not sufficient to confer processivity.

The processivity of *Ec*-PME is facilitated by four main events, the first three of which involve translocation of the substrate relative to the protein by one sugar residue, and the last (the de-methylesterification itself) that allows the cycle to repeat.

Firstly, the electrostatic repulsion between the newly created carboxylate group of the sugar residue docked in the +1 subsite and the oxy-anionic catalytic pocket generates a rotation that orients the methylester of the adjacent residue at subsite +2 towards the catalytic pocket for the next catalytic event.

Secondly, correlated motions of regions flanking the binding groove of the enzyme modulate the affinity along the groove with a sequence of expanding and constricting (breathing) movements. Such large conformational variations and the modulation of substrate affinity across a large binding interface are strategies that have previously been described in studies of other processive enzymes. Processive and non-processive structurally homologous carbohydrate-binding enzymes have been differentiated by the presence or not of loops surrounding the binding interface. In the case of the structurally related cellulases Cel6A and E2 from *Thermomononsporum fusca*, loops at the sides of the polysaccharide-binding groove of Cel6A are proposed to favor processivity by a mechanism similar to the one determined here from MD simulations for PME [30], [33]. Similarly, the structurally homologous cellulases Cel7A [34] and Cel7B [35], which are processive and non-processive respectively, differ only in the unstructured regions that surround the binding interface, regions that are present only in the case of the processive enzyme.

**Figure 9:**
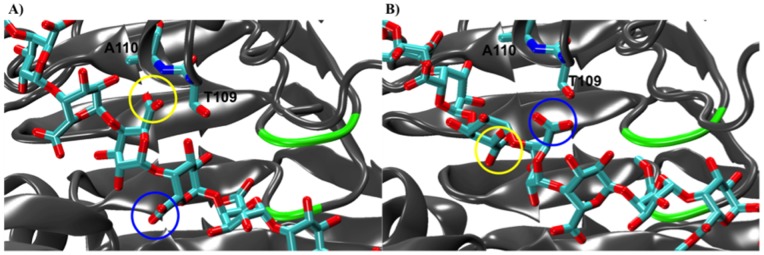
Representation of the interactions in the −2 subsite during the sliding of the decasaccharide along the binding groove of *Ec*-PME. At the start of the simulation (crystal structure coordinates) the monosaccharide residue docked in the −2 subsite establishes hydrogen bonds between Thr109 and Ala110 backbone nitrogens (A). After the rotation of the monosaccharide residues about the glycosidic bond and the sliding of the polysaccharide along the groove, the monosaccharide residue previously docked in the subsite −1 establishes contacts with Thr109 and Ala110 in the −2 subsite. The same two sugar moieties are circled in yellow and blue respectively in both (A) and (B) to highlight changes occurring in their interactions with the protein. PME secondary structure is shown in cartoon form and colored in grey. The active site (+1 subsite) region is colored in green. Thr109, Ala110 and the HG decasaccharide are represented with cyan for carbon and red for oxygen atoms.

Thirdly, the rotation of the de-methylesterified residue located in the active site, +1, also positions the carboxylate group of the galacturonic acid moiety initially docked in subsite −1 to face a positively charged patch in the −2 subsite, such that the electrostatic attraction biases the free energy landscape to encourage uni-directional motion and thus facilitate processivity. While our previous study demonstrated that the binding of a fully methylesterified chain to *Ec*-PME is possible through the establishment of hydrophobic contacts in the −2 subsite [28], un-methylesterified sugar residues in this subsite can also establish hydrogen bonds between the carboxylate oxygens of the sugar and main-chain amide atoms of Thr109 and Ala110 (Fig. 9). Interestingly, Thr109 is highly conserved among bacterial and plant PMEs, but it is absent in fungal PMEs, which are not processive. Small amino-acids that are conserved in bacterial PMEs can also be seen to have been substituted by those with larger side chains in the fungal enzymes (data not shown). The ability of *Ec*-PME to bind both methylesterified and un-methylesterified HG polysaccharides is likely to be useful in maintaining the pathogen’s capability to attack PCW pectin at different stages of plant development. Such flexibility in the binding register of the enzyme is largely determined by the ability of subsite −2 to interact with either un-methylesterified or methylesterified residues.

Fourthly, once the described translocation has successfully delivered a new methylesterified group to the active site, de-methylesterification provides the required free energy to reset the system potential, providing the ratchet required for rectification of the motion, and allowing the whole cycle to repeat. The concept of such an electrostatic ratchet has been discussed previously in a study of λ exonuclease, an enzyme that unwinds and degrades DNA [36]. In that case the biasing of the thermal fluctuations is achieved by hydrophobic interactions that impede backwards movements of the chain, and by electrostatic attractions between the DNA and the enzyme that favor the translocation of the polymer along the binding interface. A similar mechanism has been proposed for the anthrax toxin translocation [37] and is demonstrated here for PME in which enzyme-polysaccharide electrostatic interactions bias the energy landscape and facilitate the directed motion of the chain.

**Figure 10:**
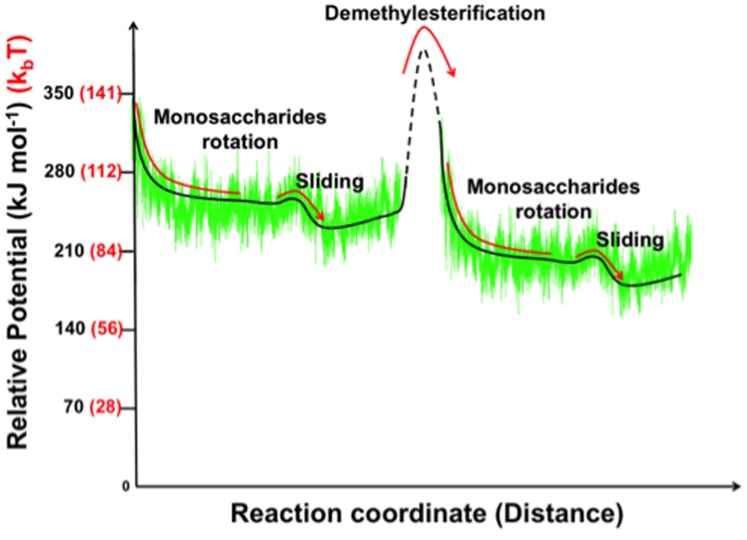
Schematic representation of the potential energy changes during *Ec*-PME processive catalysis. Calculated electrostatic binding energy of *Ec*-PME in complex to the HXM decasaccharide compared with the potential of a hypothetical molecular motor. The solid lines refer to regions of the potential sampled by molecular dynamics while the dashed line describes the final docking of the whole oligomer with the restoration of the alternating monosaccharide residue orientations and the next de-methylesterification reaction (not sampled in molecular mechanics studies). Relative potential units are reported in kJ mol^−1^ (black) and *k*_b_ *T* (red).

Figure 10 displays in schematic form the calculated electrostatic potential superimposed on a hypothetical free energy landscape inspired by standard biophysical models of molecular motors [38]. Once the next methylester group is removed the charge repulsion at subsite +1 can again drive the correlated rotations of the sugar rings, reorienting the monosaccharide subunit at +2 for processing once translated to the active site, while positioning the negatively charged residue residing at subsite −1 into a potential biased by an attracting positive charge at −2. This exquisitely orchestrated process reveals that indeed processive PMEs are uni-directional molecular motors that rectify Brownian motion by diffusing within a biased potential that is periodically lifted using the removal of pre-existing substituents on the polymer. This is in marked contrast to the use of co-factors such as ATP. While PMEs are indeed the architects of pectic fine structure, with all the functionality that that implies, pectic fine structures are, in their turn, the highways that not only direct but also power PMEs on their journeys.

Considering these processive plant and bacterial PMEs as molecular machines offers a perspective that we believe can be widely and usefully applied to many other processive enzymes.

## Methods

### Molecular Modelling and Molecular Dynamics Simulations

Noting the binding preferences found in the crystal structures, molecular dynamics (MD) simulations were performed on a complex between *Ec*-PME and a partially methylesterified HG decasaccharide with un-methylesterified residues in the subsites −5 to +1 and methylesterified residues in the subsites +2 to +5 (designated HXM). The full details of the modelling and simulations on this and other HG decasaccharides are reported elsewhere [28]. This ligand, with a carboxylate moiety binding at the active site (subsite +1), is effectively a model for the newly de-methylesterified product of the catalytic reaction. The incorporation of methylester groups on residues binding at subsites +2 to +5 allows potentially processive actions of *Ec*-PME to be studied. Simulations were repeated six times using independently generated sets of velocities for each particle and the total simulation time over the six independent runs was 300 ns (50 ns x 6).

### Principal Component Analysis of MD Trajectories

The trajectory that exhibited the sliding of the oligosaccharide along the binding groove of the enzyme was analyzed using principal component analysis (PCA) in order to investigate, without bias, the presence of correlated motions. After 15 ns equilibration, a cross-correlation matrix was generated by calculating a correlation coefficient for each Cα atom of the protein. The physically relevant motions were represented by drawing an arrow on each Cα atom having a length and color corresponding to the amplitude of the motion and an orientation corresponding to the average direction. Graphical representations were created using molecular visualization software VMD [39].

### Continuum Electrostatic Analysis of MD Trajectories

A linearized version of the Poisson-Boltzmann equation was solved to yield the electrostatic energies of *Ec*-PME and the oligosaccharide in their bound and unbound states, and from the difference the net electrostatic binding energy of the complex was estimated. The AMBER force field [40] was used to set the charges and radii of the protein atoms, while the charges and radii of the oligosaccharide atoms were defined using the GLYCAM06 force-field [41], specifically parameterized for sugar molecules. The continuum electrostatic calculations were performed with the software APBS (version 1.3) [42].

A focussing approach was chosen to carry out continuum electrostatics calculations. The accuracy of numerically solving the Poisson-Boltzmann equation for molecular systems is related to the level of coarse discretization chosen to represent the system. A focussing approach involves solving the Poisson-Boltzmann equation iteratively, increasing the number of grid points at each step without changing any other parameters. The calculation starts at longer grid spacing and increases the number of grid points at each iteration so as to reach a highest probability of convergence in smallest length scale regimes.

For every picosecond of the MD simulations three calculations were performed using the coordinates of the unbound binding partners and of the molecular complex. A cubic grid of dimensions 161 Å^3^ was used with grid spacings of 200 Å, 100 Å and 75 Å for the three calculations respectively. The electrostatic potential was visualized and colored on the molecular surface of the protein using the molecular visualization software Visual Molecular Dynamics (VMD) version 1.9.1 [39].
